# Circulating IgA Antibodies Against *Fusobacterium nucleatum* Amyloid Adhesin FadA are a Potential Biomarker for Colorectal Neoplasia

**DOI:** 10.1158/2767-9764.CRC-22-0248

**Published:** 2022-11-29

**Authors:** Jung Eun Baik, Li Li, Manish A. Shah, Daniel E. Freedberg, Zhezhen Jin, Timothy C. Wang, Yiping W. Han

**Affiliations:** 1 Division of Periodontics, College of Dental Medicine, Columbia University Irving Medical Center, New York, New York.; 2 Department of Family Medicine and UVA Comprehensive Cancer Center, University of Virginia, Charlottesville, Virginia.; 3 Division of Gastroenterology and Hepatology, Weill Cornell School of Medicine, New York, New York.; 4 Division of Digestive and Liver Diseases, Vagelos College of Physicians and Surgeons, Columbia University Irving Medical Center, New York, New York.; 5 Department of Biostatistics, Mailman School of Public Health, Columbia University, New York, New York.; 6 Herbert Irving Comprehensive Cancer Center, Columbia University Irving Medical Center, New York, New York.; 7 Department of Microbiology and Immunology, Vagelos College of Physicians and Surgeons, Columbia University Irving Medical Center, New York, New York.

## Abstract

*Fusobacterium nucleatum* (*Fn*) is a gram-negative oral anaerobe and prevalent in colorectal cancer. *Fn* encodes a unique amyloid-like adhesin, FadA complex (FadAc), consisting of intact pre-FadA and cleaved mature FadA, to promote colorectal cancer tumorigenesis. We aimed to evaluate circulating anti-FadAc antibody levels as a biomarker for colorectal cancer. Circulating anti-FadAc IgA and IgG levels were measured by ELISA in two study populations. In study 1, plasma samples from patients with colorectal cancer (*n* = 25) and matched healthy controls (*n* = 25) were obtained from University Hospitals Cleveland Medical Center. Plasma levels of anti-FadAc IgA were significantly increased in patients with colorectal cancer (mean ± SD: 1.48 ± 1.07 μg/mL) compared with matched healthy controls (0.71 ± 0.36 μg/mL; *P* = 0.001). The increase was significant in both early (stages I and II) and advanced (stages III and IV) colorectal cancer. In study 2, sera from patients with colorectal cancer (*n* = 50) and patients with advanced colorectal adenomas (*n* = 50) were obtained from the Weill Cornell Medical Center biobank. Anti-FadAc antibody titers were stratified according to the tumor stage and location. Similar as study 1, serum levels of anti-FadAc IgA were significantly increased in patients with colorectal cancer (2.06 ± 1.47 μg/mL) compared with patients with colorectal adenomas (1.49 ± 0.99 μg/mL; *P* = 0.025). Significant increase was limited to proximal cancers, but not distal tumors. Anti-FadAc IgG was not increased in either study population, suggesting that *Fn* likely translocates through the gastrointestinal tract and interact with colonic mucosa. Anti-FadAc IgA, but not IgG, is a potential biomarker for early detection of colorectal neoplasia, especially for proximal tumors.

**Significance:**

*Fn*, an oral anaerobe highly prevalent in colorectal cancer, secretes the amyloid-like FadAc to promote colorectal cancer tumorigenesis. We report that circulating levels of anti-FadAc IgA, but not IgG, are increased in patients with both early and advanced colorectal cancer compared with the healthy controls, and especially in those with proximal colorectal cancer. Anti-FadAc IgA may be developed into a serological biomarker for early detection of colorectal cancer.

## Introduction

Colorectal cancer is the third-most common cancer worldwide and the fourth leading cause of cancer death (1). Many genetic and environmental risk factors have been identified for colorectal cancer such as genomic instability, gene mutations, Western diet, lifestyle, and obesity (2, 3). In addition, accumulating studies have now recognized dysbiosis of gut microbiome as a risk factor in the development and progression of colorectal cancer (4–6). Gut microbiome contributes to colorectal carcinogenesis through several mechanisms: (i) by directly inducing carcinogenesis using their virulence factors or metabolites (7–11), (ii) by altering host immune-surveillance systems (12–15), and (iii) by affecting the efficacy of anticancer therapies such as chemotherapy and immunotherapy (16–19).

Among the cancer-associated bacteria, *Fusobacterium nucleatum* (*Fn*)*,* a gram-negative oral commensal anaerobe, is considered a key pathogen implicated in colorectal cancer (20, 21). The *Fn* level is not only significantly higher in tumor tissues compared with the normal controls (22–24), it is also highly correlated with lower survival rate (25, 26), advanced cancer stages (27–29), proximal location (6, 30–32), metastasis (33, 34), and recurrence (16, 35). In addition, increased level of *Fn* has been reported to be associated with microsatellite instability, CpG island methylator phenotype, and oncogenic mutations (26, 36).

FadA is a unique adhesin highly conserved among pathogenic *Fusobacterium* species and a major virulence factor responsible for bacterial adhesion, induction of inflammation, and colorectal cancer cell proliferation (7, 11). The *fadA* gene levels are 10- to 100-fold higher in colorectal tissues of patients with adenomas or colorectal cancer compared with the normal subjects (7). It is also significantly increased in the fecal microbiome in patients with colorectal cancer compared with the controls (21). FadA consists of two forms, the intact pre-FadA and mature FadA without signal peptide, both of which constitute the active and amyloid-like FadA complex (FadAc; ref. 37). The *fadA* mRNA is constitutively expressed (38); however, amyloid FadAc is only produced under stress and disease conditions, serving as a molecular switch to convert *Fn* from a benign commensal to a pathogen (39). Amyloid FadAc binds to extracellular domain of E-cadherin leading to the proliferation of cancer cells via activation of Annexin A1 and Wnt/β-catenin signaling (7, 11).

Early diagnosis and treatment of colorectal cancer is the most important determining factor for prognosis and patient survival. Although there are several commercially available serologic biomarkers for colorectal cancer such as carcinoembryonic antigen (CEA), alpha fetoprotein (AFP), and cancer antigen (CA) 19-9 (40–42), it has become clear that these conventional biomarkers have low sensitivity and specificity and are not suited for early diagnosis and predicting prognosis of colorectal cancer (43–45). Because of the high correlation between *Fn* and colorectal cancer progression, there have been attempts to use *Fn* as a biomarker. In this study, we investigate the levels of circulating anti-FadA antibodies in the plasma and serum from two U.S. cohorts to evaluate its applicability as a novel colorectal cancer biomarker.

## Materials and Methods

### Sample Description

Samples from two existing colorectal cancer sources were used following appropriate ethical guidelines. Study 1 samples were randomly selected from the Kentucky Colon Cancer Genetic Epidemiology Study, a population-base case–control study based on the Kentucky Cancer Registry (KCR; refs. 46, 47). Recruitment of participants was conducted between April 2003 and December 2010. A total of 1,040 incident colon cancer cases and 1,750 population-based controls completed the study with the collection of comprehensive lifestyle and epidemiological data, pathology information, and fasting blood samples. Cases were defined as individuals diagnosed with histopathologically confirmed incident primary colon cancer (excluding patients with rectal or syndromic cancers) who were invited to participate in the study within 3 months of KCR registration. Cases were eligible to participate if they: (i) had a nonrecurrent diagnosis; (ii) had no known family history or diagnosis of familial adenomatous polyposis (FAP), hereditary nonpolyposis colorectal cancer (HNPCC), Peutz-Jeghers, or Cowden disease; (iii) had no known diagnosis of inflammatory bowel disease such as Crohn disease or ulcerative colitis; (iv) were at least 21 years of age at the time of diagnosis; (v) had contact information listed in the KCR database; (vi) were willing to complete two questionnaires. The majority of participants completed data collection within 12 months (median of 5 months) of their colon cancer diagnosis.

Random digit dialing and friend referrals were utilized to recruit controls representative of the general Kentucky population. Controls consisted of frequency-matched individuals who have never been diagnosed with any cancer except nonmelanoma skin cancer and are over the age of 30, preferably ≥ 50 years old. For cases and controls, self-reported inflammatory bowel disease (e.g., Crohn disease or ulcerative colitis), family history of FAP, and HNPCC were excluded in the recruitment. The response rate for cases and controls who answered the phone and allowed eligibility determination was 70.8% and 66.7%, respectively. The study was approved by the Institutional Review Boards of the University of Kentucky (Lexington, Kentucky) and University of Virginia (Charlottesville, VA). All participants provided written informed consent.

Eligible cases and controls donated one blood sample and answered self-administered questionnaires. A two-step approach was used to collect blood samples and lifestyle risk factor data. First, a prepacked phlebotomy kit with detailed written instructions for blood sample collection and written consent forms was sent to each case subject. Participants were instructed to go to their physician offices or adjacent medical facilities for blood draw after overnight fasting. The samples were collected in purple-top (K3EDTA) blood collection tubes and shipped overnight on frozen ice pack. Upon receipt, the blood tubes were spun for 15 minutes at 600 × *g* and aliquots of plasma and concentrated buffy coat were prepared and frozen at −80°C. Second, a self-administered lifestyle risk factor questionnaire developed by the NCI Colon Cancer Familial Cancer Registry was sent to each participant to collect detailed information on demographics and lifestyle risk factors. The parent Kentucky Colon Cancer Genetic Epidemiology Study is not a matched (or paired) case–control study. Deidentified plasma samples from 25 randomly selected patients with colorectal cancer and 25 controls matched by gender and age (±1 year) were tested. The patient characteristics are summarized in Table 1.

Study 2 included randomly selected patients with advanced adenoma or adenocarcinoma who underwent surgical treatment at NYP/Weill Cornell Medical Center from 2013 to 2015. All patients who were seen in the colorectal group were offered participation in the clinical trial. Tissues were collected among those patients who provided consent, and tissue acquisition was feasible. An advanced adenoma was defined as a polyp that was greater than 3 cm in size, and was unable to be removed endoscopically. Deidentified serum samples from 50 patients with adenoma and 50 patients with adenocarcinoma were tested. Tumor stages were classified according to the Union for International Cancer Control classification. The patient characteristics are summarized in Table 1. For tumor locations in study 2, “proximal” include samples labeled as cecum, ascending, ascending/transverse, right colon, or right/cecum, while “distal” include samples labeled as sigmoid, descending, sigmoid/rectum, left colon, rectosigmoid junction, rectum. Samples from patients with mixed proximal and distal tumors were excluded. No tumor location was available for study 1. The study was approved by the Institutional Review Boards of the Weill Cornell Medical Center. All participants provided written informed consent.

**TABLE 1:** Patient characteristics

Study 1 (Kentucky)		Normal (*N* = 25)	CRC (*N* = 25)	*P*
Gender	Male Female	12 (48%) 13 (52%)	12 (48%) 13 (52%)	1.0
Age	Mean (yrs) No. <65 yrs No. >65 yrs	59.7 ± 9.9 16 (64%) 9 (36%)	59.6 ± 9.2 16 (64%) 9 (36%)	0.953 1.0
Tumor stage	I+II III+IV	— —	11 (44%) 14 (56%)	
**Study 2 (NYP/Cornell)**		**Adenomas (*N* = 50)**	**CRC (*N* = 50)**	***P***
Gender	Male Female	31 (62%) 19 (38%)	18 (36%) 32 (64%)	0.009
Age	Mean (yrs) No. <65 No. >65	64.9 ± 12.6 26 (52%) 24 (48%)	67.6 ± 12.4 23 (46%) 27 (54%)	0.291 0.548
Tumor stage	I+II III+IV	— —	30 (60%) 19 (38%)	
Tumor location^a^	Proximal Distal Other	17 (34%) 11 (22%) 22 (44%)	23 (46%) 22 (44%) 5 (10%)	<0.001

Abbreviations: CRC, colorectal cancer; yrs, years.

^a^Proximal includes patients with tumors in cecum, ascending and transverse colon; Distal includes patients with tumors in descending and sigmoid colon, and rectum; Other includes patients with tumors of unclear location or in both proximal and distal colons.

### Purification of FadAc

The recombinant FadAc protein was prepared from *Escherichia coli* (*E. coli*) BL21 (DE3) carrying pYWH471-6 as described previously (37). *E. coli* BL21 (DE3) carrying pYWH471-6 was grown in Luria-Bertani broth containing 100 μg/mL ampicillin to mid-log phase followed by incubation with 0.1 mmol/L Isopropyl β-D-1-thiogalactopyranoside (Sigma-Aldrich) for 2.5 hours at 37°C to induce expression of the recombinant FadAc protein. The bacteria were harvested by centrifugation at 8,000 *g* for 5 minutes, the pellet (∼6 g) was incubated with 50 mL of buffer A (50 mmol/L NaH_2_PO_4_, 300 mmol/L NaCl, 8 mol/L Urea, pH 8.0) at 4°C overnight. After removing cells, debris, and insoluble material by centrifugation, the clear lysate was incubated with 5 mL TALON Metal Affinity Resins (Clontech Laboratories, Inc.) for 4 hours at 4°C. The mixture was transferred to a glass chromatography column (3 × 15 cm) and unbound materials were washed out with 150 mL of buffer A. The column was then eluted with 30 mL of buffer B (50 mmol/L NaH_2_PO_4_, 300 mmol/L NaCl, 8 mol/L Urea, pH 5.0) and the elute was serially collected in 3 mL aliquots. The column fraction containing recombinant FadAc were pooled and dialyzed against PBS (pH 7.2) in a dialysis tubing with MWCO of 6–8 kDa (Spectra/Por1; Spectrum Laboratories, Inc.). The concentration of purified recombinant FadA was measured by BCA protein assay kit (Thermo Fisher Scientific).

### Preparation of Monoclonal Anti-FadA IgG

The mouse monoclonal (mAb) anti-FadA IgG (5G113G8) was prepared at the Hybridoma Core (Lerner Research Institute) described previously (37) and was used as a standard for determining the concentrations of anti-FadAc IgA and IgG in the samples. This approach is feasible because FadA is highly conserved in *Fn* (48).

### ELISA

The levels of anti-FadAc IgA and IgG were determined by indirect ELISA. The 96-well ELISA plates were coated with 100 μL of purified recombinant FadAc (2 μg/mL) in 0.2 mol/L carbonate/bicarbonate buffer (pH 9.4) and incubated at 4°C overnight. After blocking, the wells were incubated with 100 μL of blood samples serially diluted in blocking buffer for 1 hour at room temperature. To obtain a standard curve, the wells were incubated with 100 μL of purified mouse mAb 5G113G8 at increasing concentrations. Each well was then incubated with 100 μL of goat anti-human IgA-HRP (1:6,000 dilution; PA1-74395, Thermo Fisher Scientific), goat-anti-human IgG-HRP (1:10,000 dilution; A2290, Sigma-Aldrich), or goat anti-mouse IgG-HRP (1:12,000 dilution; 62-6520, Thermo Fisher Scientific) for 1 hour at room temperature, followed by incubation with 100 μL substrate solution (1-Step Ultra TMB-ELISA; Thermo Fisher Scientific) for 30 minutes. The reaction was terminated with 2 mol/L H_2_SO_4_ and the absorbance at 405 nm was measured using an automated microplate reader. The levels of anti-FadAc IgA and IgG were determined by testing serial dilutions of the plasma or serum. The values that fell into the linear range of the standard curves generated using mouse mAb 5G113G8 were used to calculate the antibody concentrations. Wells with secondary antibodies alone were used to determine background values. All measurements were performed in duplicate. All assays were performed by one single individual. Intraplate and interplate coefficient of variation (CV) in study 1 and study 2 were as follow: study 1 intraplate CV = 8.2 ± 6.12%, interplate CV = 9.5 ± 0.6%; study 2 intraplate CV = 9.7 ± 6.7%, interplate CV = 10.8 ± 1.7%.

### Statistical Analysis

Categorical variables were presented as numbers and percentages and were compared using *χ*^2^ test or Fisher exact test between two groups. Continuous variables were expressed as mean ± SD and were compared using two-tailed Student *t* test between two groups. The optimal cutoffs of plasma and serum levels of anti-FadAc IgA and IgG were identified with ROC curve analysis using Youden index criterion with the requirement that sensitivity and specificity were at least 0.50. To examine the effect of colorectal cancer status in relation to the levels of anti-FadAc IgA and IgG, a multivariable linear regression model was used to adjust for confounding factor(s) (e.g., gender) with *P* value <0.1 in univariable analysis, with the status being coded as binary dummy variables. Study 1 was matched for sex and age, but not study 2. Therefore, the adjustment of gender and age was only considered for study 2. Tumor stage and location were classified into two groups. Analysis with nonparametric methods Wilcoxon rank-sum test and Kruskal–Wallis test yielded similar results. The results with *P* value <0.05 were considered significant. The analysis was carried out with SAS software version 9.4 (SAS Institute Inc.) and figures were generated using Excel. Additional analyses are available in Supplementary Appendix.

### Data Availability Statement

Data are available upon request.

## Results

### Plasma Levels of Anti-FadAc IgA, but not IgG, are Significantly Increased in Patients with Colorectal Cancer Compared with Healthy Controls

**FIGURE 1:**
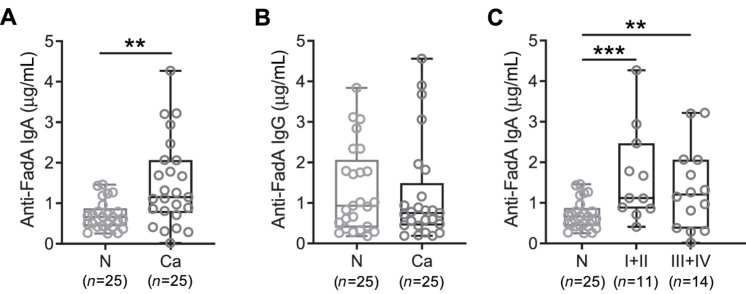
Comparison of anti-FadAc IgA or anti-FadAc IgG levels in plasma from normal subjects and patients with colorectal cancer in study 1. Anti-FadAc IgA (**A**) and anti-FadAc IgG (**B**) levels in plasma samples from 25 normal subjects (N) and 25 patients with colorectal cancer (Ca). **C,** Plasma anti-FadAc IgA levels in normal subject (N), patient with early stages of colorectal cancer (I+II), and patient with advanced stages of colorectal cancer (III+IV). Each symbol represents one human subject. Horizontal lines indicate median values, boxes show the 25th–75th percentiles, and whiskers show the minimal and maximal individual values. **, *P* < 0.01; ***, *P* < 0.001.

In study 1, the plasma levels of anti-FadAc IgA were significantly increased in patients with colorectal cancer compared with healthy subjects (1.48 ± 1.07 μg/mL vs. 0.71 ± 0.36 μg/mL, *P* = 0.001; Fig. 1A). The Youden index criterion in the ROC curve analysis yielded the cutoff of 0.81 μg/mL for anti-FadAc IgA levels, with 76% sensitivity and 76% specificity. No difference in anti-FadAc IgG was detected between the two groups (1.20 ± 1.26 μg/mL vs. 1.33 ± 1.07 μg/mL, *P* = 0.71; Fig. 1B). Furthermore, when the IgA titers were stratified by cancer stage, significant increase was detected in both early (stages I and II) and advanced (stages III and IV) colorectal cancer, compared with the normal controls (Fig. 1C). No tumor location was available for study 1.

### Serum Levels of Anti-FadAc IgA, But not IgG, are Significantly Increased in Patients with Colorectal Cancer Compared with Advanced Precancerous Polyps

**FIGURE 2:**
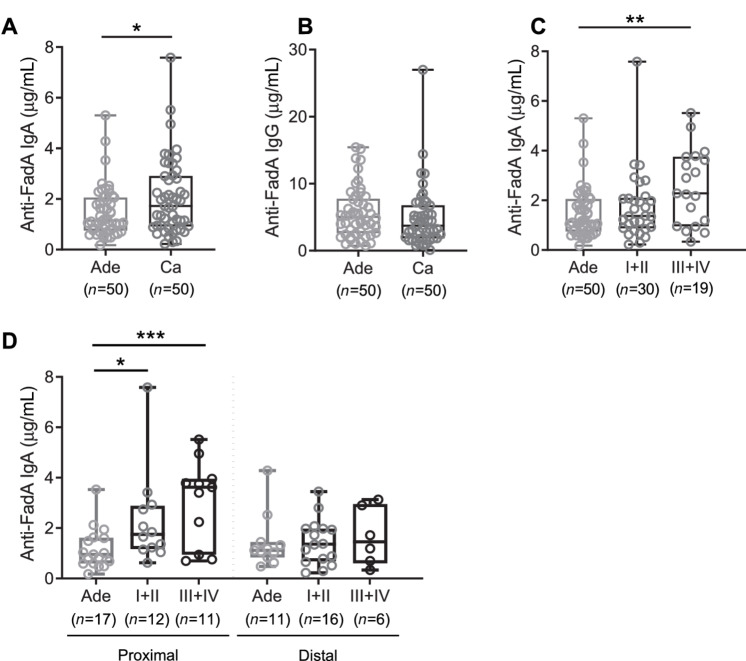
Comparison of anti-FadAc IgA or anti-FadAc IgG levels in serum from patients with advanced adenomas and colorectal cancer in study 2. Anti-FadAc IgA (**A**) and anti-FadAc IgG (**B**) levels in serum samples were obtained from 50 patients each with advanced adenomas (Ade) or colorectal cancer (Ca). **C,** Serum anti-FadAc IgA levels in patient with advanced adenomas (Ade), early stages of colorectal cancer (I+II), and advanced stages of colorectal cancer (III+IV). **D,** Serum anti-FadAc IgA levels in patients with different tumor stages [advanced adenoma (Ade), early colorectal cancer (I+II), advanced colorectal cancer (III+IV)] and tumor locations. Tumor locations were categorized into proximal (cecum, ascending, right colon) and distal (sigmoid, rectum, descending, left colon, rectosigmoid junction). Patients with tumors of unclear location or in both proximal and distal colons were excluded. Each symbol represents one human subject. Horizontal lines indicate median values, boxes show the 25th–75th percentiles, and whiskers show the minimal and maximal individual values. *, *P* < 0.05; **, *P* < 0.01; ***, *P* < 0.001.

Similarly, serum anti-FadAc IgA titers from study 2 were also found to be significantly increased in patients with colorectal cancer compared with those with advanced adenomas (2.06 ± 1.47 μg/mL vs. 1.49 ± 0.99 μg/mL, *P* = 0.025; Fig. 2A). There was a significant gender difference between the adenoma and colorectal cancer groups in study 2 (Table 1). After adjusting for gender, anti-FadAc IgA levels remain significantly higher in colorectal cancer compared with the advanced adenoma group (estimate = 0.592 μg/mL, SE = 0.262, *P* = 0.026). No difference was detected in anti-FadAc IgG levels (4.87 ± 4.46 μg/mL vs. 7.04 ± 10.7 μg/mL, *P* = 0.190; Fig. 2B).

When the results were stratified with tumor stage and location, the anti-FadAc IgA levels were only increased in advanced colorectal cancer compared to the advanced adenoma group (Fig. 2C). However, for patients with proximal tumors, the IgA levels were significantly increased in both early and advanced stages of colorectal cancer compared with the advanced adenoma group (Fig. 2D).

## Discussion

In this investigation, we found that levels of circulating anti-FadAc IgA were significantly increased in patients with colorectal cancer compared with healthy controls or to patient with advanced adenomas. The anti-FadAc IgA levels were elevated in both early and advanced colorectal cancer suggesting that it may be useful for not only advanced-stage but also early-stage diagnosis. This is consistent with our previous finding that the *fadA* genes levels are increased stepwise from normal to adenomas, and from adenomas to carcinomas (7). The adenoma group in study 2 exhibited higher titers than the normal subjects of cohort 1. However, because different specimens (plasma vs. serum) were used for these two studies, the results are not directly comparable. The elevated anti-FadAc IgA is closely associated with proximal tumors, consistent with previous reports of enriched *Fn* in proximal colorectal cancer (30, 31). Interestingly, we did not observe changes in IgG levels. These findings suggest that *Fn* likely translocates through the digestive tract inducing mucosal immune responses.

Serologic diagnosis is desirable due to the noninvasive nature, as well as easy attainment of the specimens. Previous studies reported colorectal cancer diagnosis using serum CEA, AFP, and CA19-9, achieving sensitivities of 80.43%, 73.91%, and 69.57%, and specificities of 75.00%, 69.44%, and 61.11%, respectively (41). Using anti-FadAc IgA, we achieved sensitivity and specificity both at 76%, demonstrating its superiority over AFP and CA 19-9 as potential markers. Using whole bacteria *Fn* as antigen, a previous study tested anti-*Fn* IgA in colorectal cancer, with a low sensitivity of 36.43%. Even when combined with CEA, the sensitivity was still only 53.10% (49). This may be due to the presence of common bacterial components conserved among most bacterial species such as outer membrane proteins and lipopolysaccharides making it difficult to predict colorectal cancer. Given that FadA is uniquely conserved in *Fn*, anti-FadAc antibody is a more specific biomarker. Furthermore, we used the colorectal cancer–promoting form of FadA, that is, the amyloid FadAc (39), thus achieving significantly improved sensitivity. We used the Youden index with the requirement of both sensitivity and specificity greater than 0.5 for the selection of the cut-off points. By doing so, it guarantees the cutoff yields a better approach than a random decision with a fair coin toss for potential clinical diagnosis. We anticipate that combining circulating anti-FadAc IgA with additional biomarkers may further improve the diagnostic sensitivity and specificity.

Our study had a few limitations. First, our sample size was limited, especially for those with clearly defined tumor locations. Future studies with larger sample sizes and external validations are warranted to confirm the findings, and to compare with the above-mentioned serologic parameters to stratify to additional patient and tumor characteristics, such as smoking, tumor histology, and molecular types. Second, as a proof-of-concept study, we performed retrospective analysis using samples available to us. As a result, different specimens were used in the two studies, one with plasma and the other with serum, rendering the results from the two studies incomparable. There was no adenoma group in study 1 and no healthy controls in study 2; therefore, we do not know whether anti-FadAc IgA can also be used for early detection of advanced precancerous adenomas. Third, given the retrospective nature of study, we cannot dismiss the possibility of reverse causality. As such, caution must be exercised for causal interpretation of our results. Prospective cohort study is warranted.

In summary, our study sheds novel light on using specific bacterial virulence factors as biomarkers for detection of colorectal neoplasia. Anti-FadAc IgA is a potential novel biomarker for colorectal cancer, especially for the tumors located in the proximal colon.

## Supplementary Material

Supplementary Data
