# Supplementary material for: Circulating IgA Antibodies Against Fusobacterium nucleatum Amyloid Adhesin FadA are a Potential Biomarker for Colorectal Neoplasia
Source: Cancer Res Commun. 2022 Nov 29;2(11):1497–503. doi: 10.1158/2767-9764.CRC-22-0248 (PMC10035380; doi:10.1158/2767-9764.CRC-22-0248)
Supplement: Supplementary Data [file crc-22-0248-s01.docx]

For study 1, the Box-Cox transformation shows that (IgA)^0.25^ and log(IgG) follow normal distribution, following table shows the results from analysis with and without the transformations.

**Table 1. Comparative analysis of Study 1 results with and without transformation.**

|  | CRC (n=25) | Healthy (n=25) | Two-sample t-test p-value | Wilcoxon two-sample test p-value |
| --- | --- | --- | --- | --- |
| (IgA)^0.25^ | 1.04±0.24 | 0.90±0.12 | 0.010 | 0.006 |
| log(IgG) | -0.25±0.94 | -0.07±0.91 | 0.49 | 0.46 |

|  | CRC Stage I-II (n=11) | Healthy (n=25) | Two-sample t-test p-value | Wilcoxon two-sample test p-value |
| --- | --- | --- | --- | --- |
| (IgA)^0.25^ | 1.09±0.19 | 0.90±0.12 | 0.007 | 0.007 |
| log(IgG) | -0.17±0.97 | -0.07±0.91 | 0.77 | 0.81 |

|  | CRC stage III-IV (n=13) | Healthy (n=25) | Two-sample t-test p-value | Wilcoxon two-sample test p-value |
| --- | --- | --- | --- | --- |
| (IgA)^0.25^ | 0.97±0.26 | 0.90±0.12 | 0.34 | 0.13 |
| log(IgG) | -0.35±0.97 | -0.07±0.91 | 0.38 | 0.32 |

For study 2, the Box-Cox transformation shows that log(IgA) and log(IgG) follow normal distribution, following table shows the results from analysis with and without the transformations.

**Table 2. Comparative analysis of Study 2 results with and without transformations.**

|  | CRC (n=50) | Healthy (n=50) | Two-sample t-test p-value | Wilcoxon two-sample test p-value |
| --- | --- | --- | --- | --- |
| log(IgA) | 0.48±0.77 | 0.20±0.65 | 0.049 | 0.032 |
| log(IgG) | 1.25±0.91 | 1.54±0.84 | 0.11 | 0.13 |

|  | CRC Stage I-II (n=30) | Healthy (n=50) | Two-sample t-test p-value | Wilcoxon two-sample test p-value |
| --- | --- | --- | --- | --- |
| log(IgA) | 0.34±0.07 | 0.20±0.65 | 0.36 | 0.26 |
| log(IgG) | 1.28±0.81 | 1.54±0.84 | 0.18 | 0.14 |

|  | CRC stage III-IV (n=19) | Healthy (n=50) | Two-sample t-test p-value | Wilcoxon two-sample test p-value |
| --- | --- | --- | --- | --- |
| log(IgA) | 0.70±0.77 | 0.20±0.65 | 0.008 | 0.01 |
| log(IgG) | 1.21±1.08 | 1.54±0.84 | 0.18 | 0.36 |

The tables show that the results using two-sample t-test with the data transformations are consistent with the results using nonparametric test (Wilcoxon two-sample test) with and without transformations.
